# Lost to follow-up among pregnant women in a multi-site community based maternal and newborn health registry: a prospective study

**DOI:** 10.1186/1742-4755-12-S2-S4

**Published:** 2015-06-08

**Authors:** Irene Marete, Constance Tenge, Carolyne Chemweno, Sherri Bucher, Omrana Pasha, Umesh Y Ramadurg, Shivanand C Mastiholi, Melody Chiwila, Archana Patel, Fernando Althabe, Ana Garces, Janet L Moore, Edward A Liechty, Richard J Derman, Patricia L Hibberd, K Michael Hambidge, Robert L Goldenberg, Waldemar A Carlo, Marion Koso-Thomas, Elizabeth M McClure, Fabian Esamai

**Affiliations:** 1 Department of Child Health and Paediatrics, Moi University School of Medicine, Eldoret, Kenya; 2 Department of Paediatrics, Riley Hospital, Indiana University, Indianapolis, Indiana, USA; 3 Department of Community Health Services, Aga Khan University, Karachi, Pakistan; 4 S Nijalingappa Medical College, Bagalkot, Karnataka, India; 5 K.L.E. University’s Jawaharlal Nehru Medical College, Karnataka, Belgaum, India; 6 University Teaching Hospital, Lusaka, Zambia; 7 Lata Medical Research Foundation, Maharashtra, Nagpur, India; 8 Institute for Clinical Effectiveness, Buenos Aires, Argentina; 9 Multidisciplinary Health Institute, Francisco Marroquin University, Guatemala City, Guatemala; 10 RTI International, Durham, North Carolina, USA; 11 Christiana Care Health Services, Wilmington, Delaware, USA; 12 Division of Global Health, Massachusetts General Hospital, Boston, Massachusetts, USA; 13 Department of Paediatrics, University of Colorado, Denver, Colorado, USA; 14 Columbia University, New York, New York, USA; 15 Department of Paediatrics, University of Alabama at Birmingham, Birmingham, Alabama, USA; 16 National Institute of Child Health and Human Development, Bethesda, Maryland, USA

**Keywords:** Community based registry, maternal and newborn registry, lost to follow up rate, maternal socio demographic characteristics, pregnancy outcomes

## Abstract

**Background:**

It is important when conducting epidemiologic studies to closely monitor lost to follow up (LTFU) rates. A high LTFU rate may lead to incomplete study results which in turn can introduce bias to the trial or study, threatening the validity of the findings. There is scarce information on LTFU in prospective community-based perinatal epidemiological studies. This paper reports the rates of LTFU, describes socio-demographic characteristics, and pregnancy/delivery outcomes of mothers LTFU in a large community-based pregnancy registry study.

**Methods:**

Data were from a prospective, population-based observational study of the Global Network for Women's and Children's Health Research Maternal Newborn Health Registry (MNHR). This is a multi-centre, international study in which pregnant women were enrolled in mid-pregnancy, followed through parturition and 42 days post-delivery. Risk for LTFU was calculated within a 95%CI.

**Results:**

A total of 282,626 subjects were enrolled in this study, of which 4,893 were lost to follow-up. Overall, there was a 1.7% LTFU to follow up rate. Factors associated with a higher LTFU included mothers who did not know their last menstrual period (RR 2.2, 95% CI 1.1, 4.4), maternal age of < 20 years (RR 1.2, 95% CI 1.1, 1.3), women with no formal education (RR 1.2, 95% CI 1.1, 1.4), and attending a government clinic for antenatal care (RR 2.0, 95% CI 1.4, 2.8). Post-natal factors associated with a higher LTFU rate included a newborn with feeding problems (RR 1.6, 94% CI 1.2, 2.2).

**Conclusions:**

The LTFU rate in this community-based registry was low (1.7%). Maternal age, maternal level of education, pregnancy status at enrollment and using a government facility for ANC are factors associated with being LTFU. Strategies to ensure representation and high retention in community studies are important to informing progress toward public health goals.

**Trial registration:**

Registration at the Clinicaltrials.gov (ID# NCT01073475).

## Background

Lost to follow up (LTFU) refers to subjects who were enrolled in a study or clinical trial, but who become lost at some point before all study procedures are completed and/or all data are collected. LTFU may lead to incomplete study results, which in turn can introduce bias to the trial or study, threatening the validity of the findings. Excessive numbers of LTFU subjects reduce the sample size, and hence the study’s power, leading to lowered precision of the estimates. Differential rates of important outcomes between subjects retained and those LTFU may also introduce bias to the results.

Thresholds for follow up between 60% and 80% have been recommended in cohort studies [1]. However, for epidemiologic studies of low incidence outcomes, this rate is likely to be too high, where it has been suggested that LTFU rates of <5% may have limited effect on conclusions, but the potential degree of bias increases as LTFU increased beyond 5% [2]. This is particularly true for studies of pregnancy outcomes, where stillbirths and neonatal mortality rates are generally <5% and maternal mortality is measured per 100,000 live births [2]. Pregnancy studies may also have particular challenges with obtaining high follow-up rates. For instance, subject relocation or death may make tracking the subject difficult and increase risk of LTFU [3-5]. In many low and middle income countries, such as India, it is common for women to move to their matrimonial home during late stages of pregnancy [6].

The socio-demographic characteristics of participants may also affect their retention in a study. For example, results from one investigation suggested that older patients were more likely to return to the study site compared to younger patients (15 to 39 years of age) [7]. Older patients may have more settled lifestyles, making it easier for them to incorporate clinic attendance for follow-up. Additionally, subjects with a rural and lower socio-economic status may be at higher risk of LTFU [8]. Finally, subjects may drop out of a study due to a burdensome protocol that becomes tiring [9]. Describing the characteristics of the LTFU population compared to those retained, informs the entire study of any systematic issues with the population that is LTFU. This also helps identify ways of minimizing the LTFU, and setting up of tracking systems and improved linkage to care and/or study protocols.

The Maternal and Newborn Health Registry (MNHR) of the *Eunice Kennedy Shriver* National Institute of Child Health and Human Development (NICHD) Global Network for Women's and Children's Health Research (Global Network) is an on-going multi-center study started in 2008 that aims to quantify and understand the trends in pregnancy services and maternal and newborn outcomes over time in defined, resource-limited geographic settings [10,11]. The MNHR attempts to enroll every pregnant woman at 20 weeks gestation (but enrollment can occur even after delivery) in order to record all the maternal and neonatal outcomes. Mothers enrolled in the study are residents of geographically defined clusters. Trained registry administrators (RAs) collect data at enrollment and at two times after delivery (within 7 days after birth and at day 42) to ascertain the immediate and longer-term pregnancy outcomes. Few studies have described LTFU in community-based prospective studies.

This paper reports the rates of LTFU of the enrolled mothers, describes their socio-demographic characteristics and compares the pregnancy/delivery outcomes of mothers LTFU after delivery and those that completed follow-up to day 42. This study had the following objectives: 1) to determine the proportion of mothers LTFU among the mothers enrolled in the MNHR study in the 7 Global Network sites in 6 countries; 2) to identify maternal socio-demographic and obstetric characteristics associated with LTFU; and 3) to compare neonatal and maternal outcomes for those LTFU after delivery and those who completed follow-up.

## Methods

### Study setting and design

Data were collected within a framework of a prospective, population-based observational study of the pregnancy outcomes, which is a multi-center study in seven sites in 6 countries: Argentina, Guatemala, Belgaum and Nagpur (both in India), Pakistan, Kenya and Zambia. The Global Network sites have 106 distinct geographic communities, also known as study clusters, with 300 to 500 annual births per cluster. Each site goes to great lengths to ensure that all eligible pregnant women are included in the MNHR and that all outcomes of the mothers and babies are obtained through 42 days post-partum. The methods and initial results of the MNHR have been described in detail in previous publications [10-12]. The major goal of the MNHR is to generate data to accurately estimate perinatal, neonatal, and maternal mortality in these countries. Culturally appropriate methods for tracking enrolled women in order to ensure completeness of reporting are employed in each site.

### Study population

Eligible subjects included all pregnant women living within the defined geographical boundaries of the study catchment region. For the purposes of these analyses, a woman was considered to have been enrolled into the MNHR once she gave informed consent and the initial demographic data form was completed. In all clusters, RAs enroll pregnant mothers as early as possible and follow them through delivery and to 42 days postpartum. At the time of enrollment, maternal socio-demographic and obstetric characteristics are recorded; mothers were also encouraged to seek antenatal care in the health facilities. Maternal and neonatal birth outcomes are recorded within one week after delivery, and outcomes occurring thereafter are recorded at the day 42 visit. Mothers who were enrolled and expected to complete their 6-week outcome from January 1, 2010 to December 31, 2013 were included in this analysis.

### Study procedures

Data were collected and entered at each study site and transmitted through secure methods to a central data coordinating center at Research Triangle Institute in North Carolina, USA (RTI). Retrospective data review was done to identify mothers who were LTFU. LTFU was categorized based on the point of follow-up at which the mother was lost. Some mothers were LTFU before delivery (LTFU-before). For these subjects, only basic demographic data are available. Other subjects were LTFU between delivery and the 42 day visit (LTFU-after). For these subjects, only demographic and delivery outcomes are available. Mothers missing both visits were considered to have been LTFU-before delivery.

### Data entry, edits and analysis

All analyses were performed with SAS version 9.3 (SAS Institute, Cary, NC, USA). Analyses included descriptive statistics. We modeled the risk of LTFU and calculated point and interval estimates of risk ratios using multivariable generalized linear regression log-binomial models with an exchangeable correlation matrix. We used generalized estimating equations to account for correlation of outcomes within clusters to assure appropriately sized p-values and confidence intervals.

### Ethical considerations

The Institutional Review Boards and Ethics Research Committees of the participating institutions and the Ministries of Health of the respective countries approved the MNHR. Prior to initiation of the study, approval was sought from the participating communities through sensitization meetings. Individual informed consent for study participation is requested from each study participant. No monetary reimbursements are provided to study participants nor to the communities participating in the study. A Data Monitoring Committee, appointed by the NICHD, oversees and reviews the study at annual meetings.

## Results

### Lost to follow up rates

**Figure 1:**
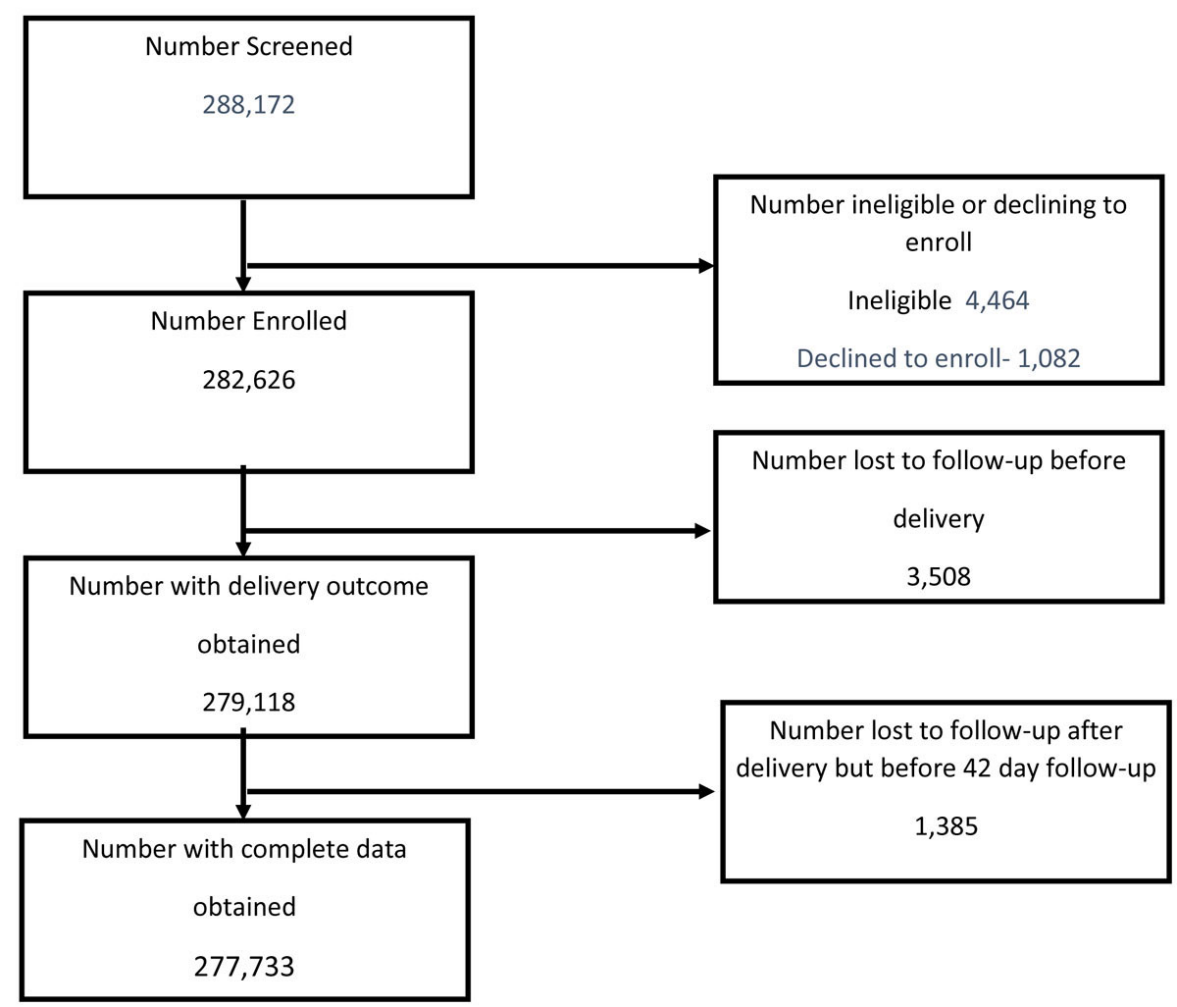
CONSORT diagram

**Figure 2:**
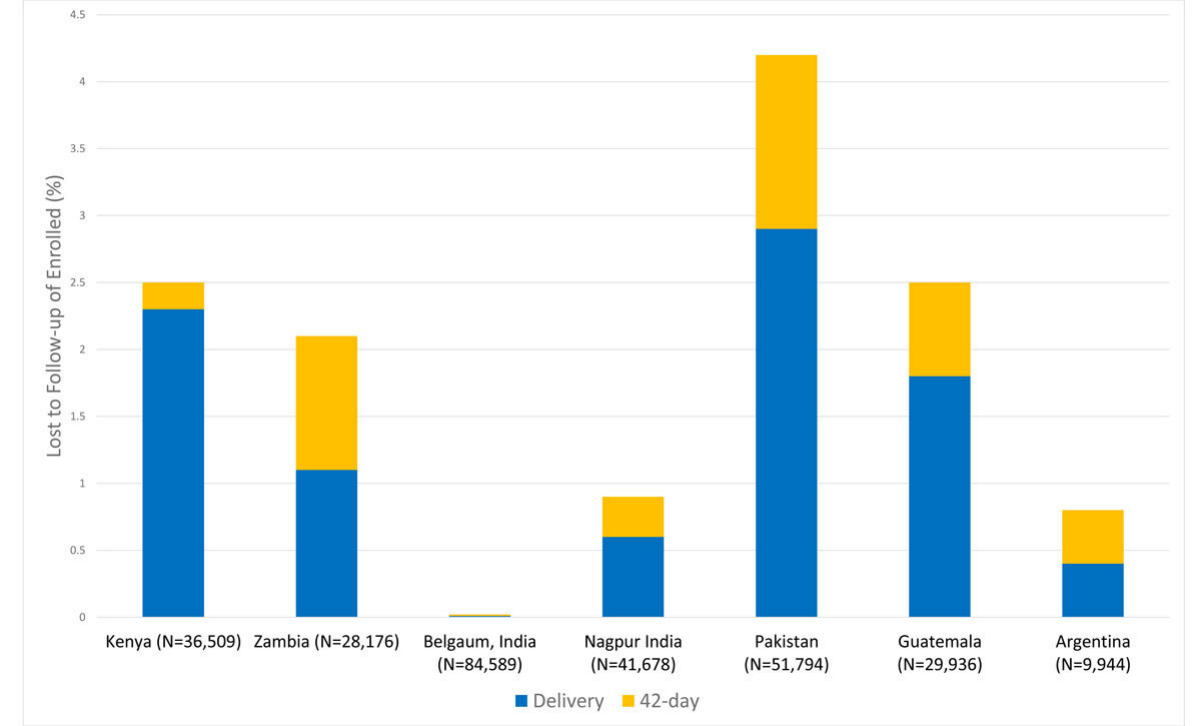
Maternal Registry: Rates of lost to follow-up at delivery and lost at day 42 postpartum by study site, 2010-2013

A total of 282,626 mothers were enrolled into the registry who expected to deliver between January 2010 and December 2013. Most of these women (87.8%) were enrolled before delivery, but 12.2% were enrolled late, either at delivery or shortly after. Among all women enrolled, 4,893 (1.7%) mothers were LTFU before post-partum day 42. Most of these were LTFU before delivery (1.2%), and hence only demographic information on the mother was available. Of those enrolled, 0.5% had data collected after delivery, but not at 42 days (Figure 1). There was variation in the number of women LTFU overall, by site, with Pakistan having the highest rate (4.2%) and Belgaum, India having the lowest (approaching 0%). The Kenya site had a large differential in LTFU rate, with 2.3% lost before delivery compared to 0.2% after delivery (Figure 2).

### Maternal characteristics

Maternal characteristics were compared for mothers who were LTFU at any point and those who completed follow–up (Table 1). Although most mothers were enrolled before delivery, those women had a higher risk of being LTFU compared to those enrolled at or after delivery (RR 2.2, 95% CI 1.7, 2.8). Those mothers who did not know their LMP had an increased risk of LTFU (RR 2.2 95% CI 1.1, 4.4). Maternal age was associated with LTFU with mothers aged <20 years having an increased risk (RR 1.2, 95% CI 1.1, 1.3). All women with less than a university education had slightly higher risks of being LTFU, and those with no formal education had the highest risk (RR 1.2, 95% CI 1.1, 1.4). Women with parity >2 were at lower risk of being LTFU than women of lower parity (RR 0.8, 95% CI 0.7, 0.9).

**Table 1:** Maternal characteristics of women in the registry by follow-up status, 2010-2013

Variable	Lost to Follow-up N (%)	Completed Follow-up N (%)	RR (95% CI)
Follow-up status, N	4,893	277,733	

Status at enrollment			

Pregnant	4,145 (96.1)	243,397 (87.7)	2.2 (1.7, 2.8)

Delivered	170 (3.9)	34,206 (12.3)	1.0

Last menstrual period known			

Yes	3,724 (76.1)	256,241 (92.3)	1.0

No	1,169 (23.9)	21,492 (7.7)	2.2 (1.1, 4.4)

Maternal age			

< 20	559 (13.0)	33,024 (11.9)	1.2 (1.1, 1.3)

20-35	3,532 (82.0)	233,647 (84.3)	1.0

> 35	214 (5.0)	10,626 (3.8)	0.8 (0.7, 1.1)

Education			

No formal education	2,090 (48.5)	69,370 (25.1)	1.2 (1.1, 1.4)

Primary	1,283 (29.8)	103,797 (37.5)	1.1 (1.0, 1.2)

Secondary	772 (17.9)	83,361 (30.1)	1.1 (1.0, 1.2)

University	162 (3.8)	19,963 (7.2)	1.0

Parity			

0	1,245 (28.9)	93,460 (33.7)	1.0 (1.0, 1.1)

1-2	1,631 (37.8)	117,243 (42.3)	1.0

> 2	1,435 (33.3)	66,217 (23.9)	0.8 (0.7, 0.9)

Last pregnancy resulted in a live birth			

Yes	2,797 (91.3)	172,183 (93.9)	1.0

No	268 (8.7)	11,164 (6.1)	1.1 (0.9, 1.3)

Table 2 shows differential health seeking behavior in those women LTFU relative to those who completed follow-up. It should be noted that these data were only available for those LTFU after delivery, roughly 28% of the total of those LTFU. Neither ANC attendance nor numbers of ANC visits were significantly associated with being LTFU; however there was a suggestion that women with no visits or fewer visits were more likely to be LTFU. Women who attended a government clinic for ANC were at significantly greater risk of being LTFU than those receiving ANC at most other locations (RR 2.0, 95% CI 1.4, 2.8).

**Table 2:** Antenatal health seeking behaviors of mothers lost to follow-up prior to 42 days after delivery compared to those who completed follow-up, 2010-2013

Variable	Lost to Follow-up N (%)	Completed Follow-up N (%)	RR (95% CI)
Follow-up Status after Delivery, N	1,385	277,733	

At least one ANC visit	1,366	277,088	

Yes	1,250 (91.5)	266,411 (96.1)	1.0

No	116 (8.5)	10,677 (3.9)	1.2 (0.8, 1.7)

Number of ANC visits	507	150,076	

0	28 (5.5)	4,588 (3.1)	1.5 (0.9, 2.5)

1-2	137 (27.0)	31,136 (20.7)	1.2 (1.0, 1.5)

≥ 3	342 (67.5)	114,352 (76.2)	1.0

Trimester for first ANC visit	1,236	259,934	

First	382 (30.9)	114,461 (44.0)	1.0

Second	515 (41.7)	100,237 (38.6)	1.0 (0.9, 1.2)

Third	339 (27.4)	45,236 (17.4)	1.1 (0.9, 1.3)

Most frequent location of ANC	1,185	247,686	

Government hospital	279 (23.5)	108,983 (44.0)	1.0

Private hospital	22 (1.9)	25,197 (10.2)	0.9 (0.8, 1.1)

Government clinic	225 (19.0)	33,831 (13.7)	2.0 (1.4, 2.8)

Private clinic	435 (36.7)	33,327 (13.5)	1.4 (0.9, 2.0)

Health worker	61 (5.1)	25,699 (10.4)	1.2 (1.0, 1.6)

Traditional birth attendant	49 (4.1)	10,125 (4.1)	1.5 (0.8, 2.7)

Other	114 (9.6)	10,524 (4.2)	2.0 (1.3, 3.1)

Tetanus toxoid vaccine	1,363	277,239	

Yes	946 (69.4)	235,088 (84.8)	1.0

No	417 (30.6)	42,151 (15.2)	1.1 (0.9, 1.4)

Prenatal vitamins/iron	1,364	277,115	

Yes	1,101 (80.7)	247,749 (89.4)	1.0

No	263 (19.3)	29,366 (10.6)	1.0 (0.8, 1.3)

HIV test	1,363	276,501	

Yes	602 (44.2)	202,164 (73.1)	1.0

No	761 (55.8)	74,337 (26.9)	1.3 (0.9, 1.7)

Among the mothers who were lost to follow-up after delivery, there were few statistically significant differences in risk of being LTFU based on the place or mode of delivery or birth attendant (Table 3). However, women who delivered at a clinic (RR 1.1, 95% CI 1.0, 1.3) or were delivered by a nurse/midwife or other health worker were slightly more likely to be LTFU (RR 1.1, 95% CI 1.0, 1.2). Neonatal outcomes are shown in Table 4. While some of the results were not statistically significant, in general women with worse outcomes were more likely to be LTFU by 42 days. This was especially apparent for women with infants with reported feeding problems (RR 1.6, 95% CI 1.2, 2.2).

**Table 3:** Delivery characteristics of mothers lost to follow up prior to 42 days after delivery, 2010-2013

	**Lost to Follow-up** N (%)	**Completed Follow-up** N (%)	RR (95% CI)
Lost to Follow-up Status After Delivery, N	1,385	277,733	

Delivery location			

Hospital	451 (33.3)	126,606 (45.6)	1.0

Clinic	394 (29.1)	68,689 (24.7)	1.1 (1.0, 1.3)

Home/Other	508 (37.5)	82,303 (29.6)	1.0 (0.8, 1.1)

Delivery mode			

Vaginal	1,110 (82.1)	231,099 (83.2)	1.0

Vaginal assisted	40 (3.0)	4,161 (1.5)	1.1 (0.6, 1.8)

C-section	154 (11.4)	33,052 (11.9)	1.0 (0.9, 1.2)

Birth attendant			

Physician	384 (28.4)	106,834 (38.5)	1.0

Nurse/Midwife/Health worker	437 (32.3)	88,316 (31.8)	1.1 (1.0, 1.2)

Traditional birth attendant	450 (33.3)	63,892 (23.0)	1.0 (0.8, 1.2)

Family/Other	80 (5.9)	18,593 (6.7)	0.9 (0.8, 1.1)

**Table 4:** Neonatal outcomes; lost to follow up prior to 42 days after delivery vs. completed follow-up

Outcome	**Lost to Follow-up** N (%)	Completed Follow-up N (%)	RR (95% CI)
Neonatal status at birth, N	1,417	280,095	

Macerated Stillbirth	20 (1.4)	2,336 (0.8)	1.3 (0.9, 2.0)

Fresh Stillbirth	37 (2.6)	4,835 (1.7)	1.3 (0.9, 1.8)

Born alive, died before visit	42 (3.0)	5,021 (1.8)	1.4 (1.0, 1.9)

Born alive, alive at visit	1,268 (89.5)	257,893 (92.1)	1.0

Congenital anomaly			

Yes	7 (0.5)	1,450 (0.5)	0.9 (0.5, 1.5)

No	1,344 (99.5)	268,544 (99.5)	1.0

Breathing problems			

Yes	117 (8.5)	13,551 (5.0)	1.1 (0.9, 1.5)

No	1,258 (91.5)	259,378 (95.0)	1.0

Feeding problems, N (%)			

Yes	38 (2.8)	4,131 (1.5)	1.6 (1.2, 2.2)

No	1,337 (97.2)	268,722 (98.5)	1.0

High fever, N (%)			

Yes	22 (1.6)	2,109 (0.8)	1.4 (0.7, 2.6)

No	1,351 (98.4)	270,892 (99.2)	1.0

Hypothermia, N (%)			

Yes	9 (0.7)	1,277 (0.5)	1.0 (0.5, 1.8)

No	1,357 (99.3)	270,345 (99.5)	1.0

Convulsions, N (%)			

Yes	1 (0.1)	452 (0.2)	0.3 (0.0, 2.7)

No	1,371 (99.9)	272,437 (99.8)	1.0

**Figure 3:**
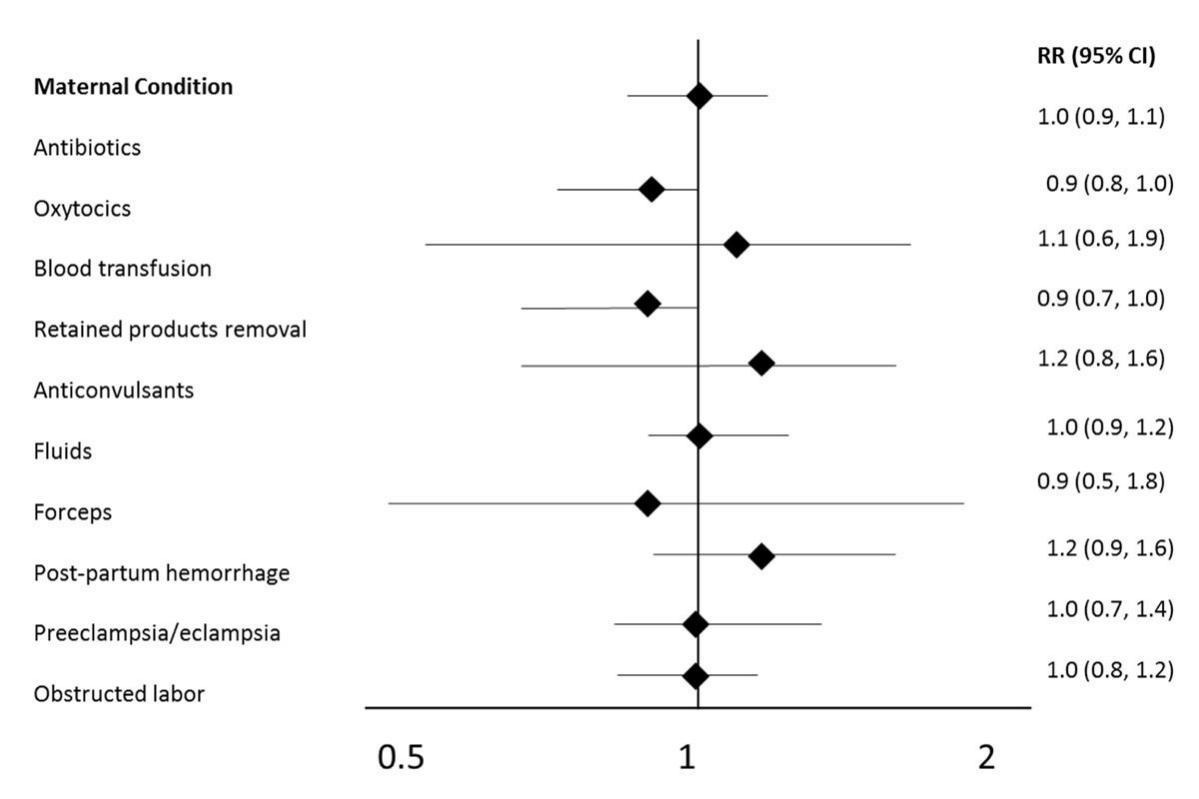
Relative risk (95% CI) of being lost to follow-up by maternal condition, 2010-2013

The associations between major maternal complications and LTFU are shown in Figure 3. There were no statistically significant differences between any of these maternal outcomes and follow-up status. Neonatal outcomes are shown in Table 4; there were also no statistically significant differences in neonatal outcomes associated with follow-up outcomes.

## Discussion

The 42-day LTFU rate in the MNHR was low, at 1.7%, in this community-based pregnancy registry study. The complete follow up rates of 98.3% surpasses by far the recommended follow up thresholds of between 60-80 percent for cohort studies [1]. However, it must be mentioned that the outcomes of interest are of relatively low incidence, so a very low LTFU rate is crucial, as missing a single rare adverse outcome disproportionately affects the conclusions. A meta-analysis of several antiretroviral therapy (ART) programs in resource limited settings showed that an average of 21% of patients had been LTFU in the first six months after starting ART [3]. These programs, however, were implemented in a clinic setting where clients were enrolled and followed up within a health facility. Long waiting lines, lack of confidentiality and privacy may have affected clients’ willingness to return for follow-up in clinic settings [4,12,13]. This is unlikely to occur in community-based registries, such as the MNHR, where follow-up is typically done in the participant’s home by the RA. However, some MNHR follow-up activities also took place where women received antenatal and postpartum care. The fact that women who most frequently utilized a government hospital or clinic for ANC also showed higher rates of LTFU may be explained by the type of care received at those locations and deserves additional investigation.

In studies like the present, some subjects are not able to be located by the study personnel. Some pregnant mothers may leave their matrimonial homes and return to their birth homes and remain there until they deliver by custom or for economic necessity. This movement has been a commonly reported phenomenon in Indian and African studies of pregnant women [5,7] and the paper in this supplement from India also documents a very high rate of movement for delivery [6]. Such migration contributes to LTFU, especially if a tracking mechanism is not in place to capture out of area births. Other potential reasons for a subject not being located include incorrect addresses or insufficient information in study logs regarding subjects’ places of residence. This is especially true in studies conducted in rural areas of low-income countries, where few subjects have a physical street address. Reliable phone contacts, head of household’s name and a notation of a landmark close to where the subject lives improve the chances of locating the participant for follow up [8].

While none of the sites had a large LTFU rate, Pakistan’s LTFU rate of 4.2% was higher than that of the other sites and considerably higher than the Indian sites and especially Belgaum’s LTFU rate. By virtually any measure of the health care system or women’s education, Pakistan performs poorly compared to the other Global Network sites, while in Belgaum, every effort is made to track women and ensure that they receive appropriate care. These differences, documented in 2 papers in this supplement, likely explain the differences in the LTFU rate between these sites [6,10].

Various techniques were employed in the different sites to ensure retention of mothers in the registry. For instance, the Kenya site gave mobile phones to village elders to improve communication between the village elders and MNHR RAs [11]. The elders were also provided with platform weighing scales to weigh all babies delivered in their villages. This culturally appropriate method to engage the village elders in the study improved the Kenya LTFU rate, and ensured that all mothers who delivered at home had accurate infant weight recorded. In addition, the village elders assisted the study personnel in contacting mothers as soon as possible after delivery [11]. In Belgaum, India, the site has worked closely with the Ministry of Health and the existing community health workers to conduct monthly visits to check on the mother’s status and track resident women who leave the area to give birth in a matrimonial home outside the cluster [6]. As another example, the Guatemala team has engaged the traditional birth attendants to help increase community involvement with the MNHR and complete follow-up visits at home for women who did not traditionally access the formal health care services.

More subjects were LTFU before delivery than after delivery. This finding was consistent in all sites except Argentina, which had an equal proportion. This means that only basic maternal demographic data were available for the majority of subjects LTFU. Some parallels exist between the results of our study and observational data on ANC clinic attendance. In most countries, the percentage of mothers who attend at least one ANC clinic is high but subsequent appointments are often not kept. Furthermore, about 20% of women who attend ANC in sub-Saharan Africa and south Asia do not seek skilled birth attendance [14]. This high rate of drop-out from the professional health care system, especially at the point of delivery, is consistent with the findings of this community registry study.

Maternal characteristics that increased risk of LTFU include: being pregnant at time of enrollment rather than enrolling at or soon after delivery, younger age (<20 years) and having no formal education. Mothers who did not know their LMP were also at increased risk of being LTFU. Unknown LMP may in some cases be associated with a pseudo pregnancy which, although rare, results in study drop-out and may also make tracking based on expected delivery more difficult [15]. Low birth weight and low socioeconomic status are also associated with an unknown LMP, as well as poor pregnancy outcome. Therefore, disproportionate loss of subjects with these characteristics are likely to bias the conclusions of a study such as this in the direction of an underestimation of neonatal and/or maternal mortality. Women’s education level has also been associated with antenatal care coverage in some studies with having a higher education level increasing the likelihood of ANC attendance and reducing the rate of LTFU [12]. Women from urban settings are also more likely to make more ANC visits compared to women from rural settings. Women who presented late (>20 weeks gestation) for ANC were twice as likely to become lost prior to delivery compared to those who presented earlier. Late presentation has been attributed to confusion over pregnancy status, fear of HIV testing, transportation limitation, lack of perceived benefits and clinic booking delays [16,17].

Other studies have found age to be associated with follow-up status where older patients were more likely to return to the clinic compared to younger patients [8]. Young mothers who become pregnant while still living with parents are likely to relocate during the study, either to get married and live with the husband, to go back to school, or to seek employment in distant major towns. Older patients may have more settled lifestyles making it easy for them to incorporate follow–up visits.

The neonatal and maternal outcomes for mothers LTFU after delivery and those who completed follow up showed no significant difference in the initial follow up. From the findings of this study, most children born to mothers LTFU after delivery were alive at the perinatal visit (89.5%). This indicates that being LTFU was not associated with adverse neonatal outcomes. This remained so even when comparison was made between sites with relatively high and those with relatively low LTFU rates. Likewise, the maternal outcomes did not differ significantly by the follow up status. It is however difficult to make a definitive comment on the outcomes as the mothers who were LTFU before delivery had no documented maternal or neonatal outcomes. Similarly those who were LTFU after delivery had no day 42 outcomes.

This study has several limitations. Clearly, the major limitation is the fact that for approximately 70% of women LTFU, no data beyond the most basic demographic indicators were available. Information on how the circumstances and immediate outcome of delivery were only available on the approximately 30% of subjects LTFU between delivery and the final 42 day outcome. It is also not known how many out of those reported as LTFU are still alive. However, it seems likely that since a maternal death is a relatively rare event, even in low and middle income countries, neighbors and village elders who were contacted would have reported any such deaths.

In conclusion, our data show that a community-based prospective birth registry can be constructed and conducted in low and middle income countries with a very low LTFU rate. Hence we are reasonably confident in the estimates of both maternal and perinatal/neonatal mortality rates. However, we must caution that there could have been a selection bias in the subjects lost to follow-up, resulting in an underestimation of mortality rates. Several risk factors emerge for LTFU, such as low maternal age and lack of education. Therefore, we recommend that special emphasis be made for subjects at risk, and tracking of these subjects be intensified during the study. Ultimately, ensuring complete representation in community-based studies such as the MNHR is needed to ensure generalizability of study findings for public health research.

## List of abbreviations used

ANC: Antenatal Care; LTFU: Lost to Follow Up; MNH: Maternal and New-born Health; RA: Registry Administrator; RTI: Research Training Institute; SVD: Spontaneous Vaginal Delivery.

## Competing interests

The authors declare that they have no competing interests.

## Author’s contributions

IM was the primary author and wrote the first draft; IM, CT, PG, HM, PN OP, SSG, M, AP, AG, FA, EAL, RJD, PLH, KMH, RLG, WAC, EMM, MKT and FE are co-investigators in the MNHR and participate in study monitoring; FE, IM, CT, SB, EAL, RLG and EMM participated in the manuscript writing. JLM conducted the data analyses. All authors reviewed the final manuscript

## Peer review

Reviewer reports for this article can be found in Additional file 1.

## Supplementary Material

Additional file 1 Click here for file

## Declarations

This article has been published as part of *Reproductive Health* Volume 12 Supplement 2, 2015: Research reports from the NICHD Global Network for Women’s and Children’s Health Research Maternal and Newborn Health Registry. The full contents of the supplement are available online at http://www.reproductive-health-journal.com/supplements/12/S2. Publication of this supplement was supported by grants from the Eunice Kennedy Shriver National Institute of Child Health and Human Development to RTI International.
