# Supplementary material for: Lost to follow-up among pregnant women in a multi-site community based maternal and newborn health registry: a prospective study
Source: Reprod Health. 2015 Jun 8;12(Suppl 2):S4. doi: 10.1186/1742-4755-12-S2-S4 (PMC4464022; doi:10.1186/1742-4755-12-S2-S4)
Supplement: Additional file 1 [file 1742-4755-12-S2-S4-S1.pdf]

Referee's comments to the authors– this sheet WILL be seen by the author(s) and published with the article

Title	Loss to follow-up among pregnant women in a multi-centre community based, maternal and newborn health registry: A prospective study
Author(s)	Irene Marete, Constance Tenge, Carolyne Chemweno, Sherri Bucher Omrana Pasha, Shivaprasad S Goudar, Musaku Mwenche, Archana Patel, Ana Garces, Fernando Althabe, Janet L Moore, Edward ALiechty, Richard J Derman, Patricia L Hibberd, K Michael Hambidge, Robert L Goldenberg, Waldemar A Carlo, Marion Koso-Thomas, Elizabeth M McClure, Fabian Esamai
Referee's name	Deborah Armbruster

When assessing the work, please consider the following points, where applicable:

1. Is the question posed by the authors new and well defined?
2. Are the methods appropriate and well described, and are sufficient details provided to replicate the work?
3. Are the data sound and well controlled?
4. Does the manuscript adhere to the relevant standards for reporting and data deposition?
5. Are the discussion and conclusions well balanced and adequately supported by the data?
6. Do the title and abstract accurately convey what has been found?
7. Is the writing acceptable?

Please make your report as constructive and detailed as possible in your comments so that authors have the opportunity to overcome any serious deficiencies that you find and please also divide your comments into the following categories:

- Major Compulsory Revisions (which the author must respond to before a decision on publication can be reached)
- Minor Essential Revisions (such as missing labels on figures, or the wrong use of a term, which the author can be trusted to correct)
- Discretionary Revisions (which are recommendations for improvement but which the author can choose to ignore)

Where possible please supply references to substantiate your comments.

When referring to the manuscript please provide specific page and paragraph citations where appropriate.

General comments: Initially, I wasn't sure that this was a topic worthy of a paper but the authors have convinced of the practical value of understanding the data behind patients/women who are "lost to follow-up". The topic is well defined, the methods appropriate and well described with sufficient details and the title describes the article well. The remaining questions listed above are properly addressed. The author(s) writes well and the article is very readable.

Major compulsory revisions:

Minor essential revisions:

Discretionary revisions:

It would be useful to create a text box with key findings that should inform others on the topic of LTFU. The abstract doesn't include all key findings and you have to wade through the article to try to find all of them.

The variations between countries, particularly Belgaum, India and Pakistan appear to be large. It would be very useful to provide information on the reasons or potential reasons for these differences. What are the differences in the sites or the differences in implementation that might account for the differences?

(continue on the next sheet)

Continued:

Have or will the sites incorporate the authors' findings into their ongoing implementation? Given that this is a large, ongoing multi-center trial, one would hope that the sites will make changes in their implementation to incorporate the author's findings. If so, or even if there is not this expectation, it would be very interesting to repeat this review of LTFU data in 2 years to see if the LTFU findings were used by the sites to make changes in their implementation to decrease LTFU -- did the study sites focus on the women at high risk of LTFU to try to decrease the LTFU, and most importantly, did it make a difference in actually decreasing LTFU? I do know that the challenge is that your LTFU is already quite small.

There are types or missing words or suffixes on pg. 7, 3rd line Maternal characteristics (missing the word "to"); pg. 9, second line, need to add, "ly" to unlike; pg. 10, first line of last paragraph, "enrollment" is misspelled

Referee's comments to the authors– this sheet WILL be seen by the author(s) and published with the article

Title	Loss to follow-up among pregnant women in a multi-centre community based, maternal and newborn health registry: A prospective study
Author(s)	Irene Marete, Constance Tenge, Carolyne Chemweno, Sherri Bucher Omrana Pasha, Shivaprasad S Goudar, Musaku Mwenche, Archana Patel, Ana Garces, Fernando Althabe, Janet L Moore, Edward ALiechty, Richard J Derman, Patricia L Hibberd, K Michael Hambidge, Robert L Goldenberg, Waldemar A Carlo, Marion Koso-Thomas, Elizabeth M McClure, Fabian Esamai
Referee's name	Jennifer Griffin

When assessing the work, please consider the following points, where applicable:

1. Is the question posed by the authors new and well defined?
2. Are the methods appropriate and well described, and are sufficient details provided to replicate the work?
3. Are the data sound and well controlled?
4. Does the manuscript adhere to the relevant standards for reporting and data deposition?
5. Are the discussion and conclusions well balanced and adequately supported by the data?
6. Do the title and abstract accurately convey what has been found?
7. Is the writing acceptable?

Please make your report as constructive and detailed as possible in your comments so that authors have the opportunity to overcome any serious deficiencies that you find and please also divide your comments into the following categories:

- Major Compulsory Revisions (which the author must respond to before a decision on publication can be reached)
- Minor Essential Revisions (such as missing labels on figures, or the wrong use of a term, which the author can be trusted to correct)
- Discretionary Revisions (which are recommendations for improvement but which the author can choose to ignore)

Where possible please supply references to substantiate your comments.

When referring to the manuscript please provide specific page and paragraph citations where appropriate.

General comments:
Loss to follow up is a problem in cohort studies and frequently leads to biased effect estimates. Examination of the magnitude and patterns of loss to follow up is important to understanding the potential for and possible direction of bias. This manuscript reports loss to follow-up, associations with maternal characteristics, and associations with maternal/neonatal health outcomes among ~280,000 pregnant women enrolled in the Global Network registry between Jan 2010 and Dec 2013. This manuscript makes an important contribution to LTFU literature in both pregnancy and community-based cohorts.

Major compulsory revisions:
METHODS:
- Data entry and edits are not discussed. Please include a brief description.
- Analysis description is inadequate for determining statistical methods used (i.e. if I had the dataset, I would not be able to replicate your analysis). Specifically:
  - What GLM did you use to estimate RRs?
  - For GEE, what correlation structure did you assume? Why?
  - How were variables selected for model inclusion/exclusion?

(continue on the next sheet)

Continued:
RESULTS:

- Please include a flow chart of subjects through the study cohort at each stage (Fig 1) to accompany text in the description of loss to follow up.
- Good presentation of raw data and CI reporting. For all tables reporting RRs, please include unadjusted RRs, as well as adjusted RRs (with description of control variables included in the model in text and in table footnote). The addition of unadjusted RRs allows one to hand calculate a few RRs and get a feel for the data. Also, allows the comparison of unadjusted and adjusted RRs, including the magnitude and direction of the change. For adjusted analyses, please include the model N, because may be different than the unadjusted analysis due to missing variables.

DISCUSSION:

- Better discussion of potential for selection bias due to LTFU is needed.
  - Low rate of LTFU is encouraging, but this study has rare outcomes, so need a caveat.
  - Paragraph 5: LMP may be associated with pseudo pregnancy (refs?), but is also associated with LBW and SES, which are associated with preterm. Unknown LMP is associated with LTFU and has the potential to introduce bias into studies of preterm. In fact, all of the risk factors for LTFU (young age, no formal education) are associated with poor perinatal outcomes and are likely to be associated with exposure variables as well and have the potential to introduce selection bias.
  - Paragraph 7. Neonatal and maternal outcomes. Although absolute numbers are low and (mostly) non-significant, there is a trend toward NN mortality, which should be addressed with appropriate caveats. Again, a crude RR would be helpful, as adjusted analyses need to be interpreted with caution.
  - Comment on expected direction of bias.

Minor essential revisions:
GENERAL COMMENTS:

- Report number of study participants in results section of abstract.
- While this manuscript is generally well-written, it would benefit from editing to address grammatical/punctuation issues
  - Write out numbers <10 in text
  - Don't start a sentence with a number
  - Loss vs lost in various places

BACKGROUND:

- Paragraph 2: If rates (20-40%) are too high for rare outcome cohort studies, what are more reasonable targets?

RESULTS:

- Subsections should match objectives outlined in Background – 1) Loss to follow up 2) Maternal characteristics 3) Neonatal and maternal outcomes
- I would prefer if Figure 2 (maternal outcomes) was also presented in a table, like all other associations.

DISCUSSION:

- Good discussion of LTFU and in this and other settings and how low LTFU was achieved in this study
- Last paragraph: "Our data suggest that the earlier in pregnancy contact is made with the subject…"- is this reported in the results? If not, add. If yes, highlight result. Also, please include a sentence about selection bias in the last paragraph.

Discretionary revisions:
BACKGROUND:

- Delete paragraph 1, sentence 2 (also in abstract). This is a confusing sentence and is redundant.
- Paragraph 1, sentence 4: I think that the key issue here is a loss of study power, which negatively impacts the precision of effect estimates vs the issue of reliability of data
- Paragraph 2, sentence 5: "Loss to follow-up"
- Paragraph 3: last two sentences should be revised for clarity
- Paragraph 4: RAs not RA's

Supplement Editor's comments

Please delete the second sentence of the Discussion: "No other community-based studies had data assessing LTFU rates that were available for comparison." To make such statement you will require to provide a systematic review of the literature.

In references not coming from Journals or books please provide the link and date accessed.

Please provide a more explanatory legend for figure 2. Each figure and it legend should be self-explanatory.
